# Gut microbes modulate (p)ppGpp during a time-restricted feeding regimen

**DOI:** 10.1128/mbio.01907-23

**Published:** 2023-11-16

**Authors:** Amy Ontai-Brenning, Randy Hamchand, Jason M. Crawford, Andrew L. Goodman

**Affiliations:** 1 Microbial Sciences Institute, Yale University, West Haven, Connecticut, USA; 2 Department of Microbial Pathogenesis, Yale University School of Medicine, New Haven, Connecticut, USA; 3 Department of Chemistry, Yale University, New Haven, Connecticut, USA; 4 Institute of Biomolecular Design & Discovery, Yale University, West Haven, Connecticut, USA; University of Hawaii at Manoa, Honolulu, Hawaii, USA; University of Oregon, Eugene, Oregon, USA

**Keywords:** gut microbiota, *Bacteroides*, fasting, (p)ppGpp, stringent response

## Abstract

Mammals exhibit daily fasting and feeding patterns that produce a fluctuating environment in the gut. By colonizing germfree mice with *Bacteroides thetaiotaomicron* and examining gene expression through a time-restricted feeding cycle, we demonstrate that this prominent gut commensal exhibits gene expression patterns characteristic of a stringent response and increases ppGpp levels during the host-fasting phase of the feeding regimen. Mutants unable to produce (p)ppGpp fail to produce this transcriptional response, exhibit unrestrained chromosomal replication, and cannot maintain population size during the host-fasting phase. Additionally, *B. thetaiotaomicron* requires (p)ppGpp to utilize host glycans both *in vitro* and *in vivo*, and mutants unable to produce (p)ppGpp display deficiencies in mucus layer colonization during the host-fasting phase. Complete gut microbial communities from mice and humans also increase ppGpp levels in this manner, demonstrating that this response appears to be general across species and conserved across mammalian gut communities. Together, these results identify an intracellular signal that allows gut microbes to coordinate their physiology with a time-restricted feeding regimen of their host.

**IMPORTANCE:**

Mammals do not eat continuously, instead concentrating their feeding to a restricted portion of the day. This behavior presents the mammalian gut microbiota with a fluctuating environment with consequences for host-microbiome interaction, infection risk, immune response, drug metabolism, and other aspects of health. We demonstrate that in mice, gut microbes elevate levels of an intracellular signaling molecule, (p)ppGpp, during the fasting phase of a time-restricted feeding regimen. Disabling this response in a representative human gut commensal species significantly reduces colonization during this host-fasting phase. This response appears to be general across species and conserved across mammalian gut communities, highlighting a pathway that allows healthy gut microbiomes to maintain stability in an unstable environment.

## INTRODUCTION

The mammalian gut microbiota comprises one of the densest known microbial communities, reaching levels of 10^11^–10^12^ cells per milligram (1). These microbes compete for limited nutrients in a fluctuating environment, where periodic waves of nutrients enter and exit the digestive tract in accordance with the feeding patterns of the host (2, 3). Both mice and humans do not eat continuously, instead concentrating feeding to a restricted portion of the day (primarily daytime for humans, primarily night for mice) (4–7). Previous research indicates that fasting and feeding cycles affect gut microbiota community composition, metabolite production, and adherence to epithelial cells (8–11). Such outcomes have important implications for human health: for example, host fasting and feeding cycles modulate the production of antimicrobial peptides in a microbiota-dependent manner (12) and fasting enhances colonization resistance to pathogens (13). While these studies indicate that the microbiota senses and responds to host eating (10), how resident gut microbes coordinate their physiology with host feeding and fasting is largely unexplored.

Periodic feeding impacts the range of nutrients available to gut microbes. Gut bacteria possess the ability to utilize many different nutritional sources, including a wide array of complex polysaccharides and fibers. For example, members of the genus *Bacteroides* (the most prominent genus in the gut of many individuals) typically devote 20% of their genomes to polysaccharide utilization loci (PULs) which are involved in sensing and degrading diverse carbohydrates (14). This allows human gut *Bacteroides* and their relatives to metabolize a wide variety of food sources, including diet-derived glycans that largely escape host digestive enzymes, and host-derived mucus glycans that line the gut lumen (14, 15).

The bacterial response to starvation and nutrient availability has been most widely explored in laboratory studies of *Escherichia coli* and *Bacillus subtilis*. Under laboratory conditions, these species and others adapt to nutrient starvation through the intracellular molecules guanosine-5′−3′-bispyrophosphate (ppGpp) and guanosine pentaphosphate (pppGpp), collectively referred to as (p)ppGpp (16–18). In response to nutrient deprivation and other stresses, bacteria produce (p)ppGpp, which, in turn, leads to a repression of growth and induction of stasis through multiple transcriptional and post-transcriptional mechanisms (16–19). This “stringent response” allows bacteria to allocate resources away from protein production in the absence of necessary resources for growth, in some cases activating alternate pathways. In a classic example, *E. coli* responds to amino acid starvation in laboratory culture by producing (p)ppGpp, which, in turn, represses expression of tRNAs and other translational machinery and activates amino acid biosynthetic pathways (20, 21). (p)ppGpp^0^ strains, which lack (p)ppGpp biosynthetic enzymes and are unable to activate this response, fail to survive amino acid starvation (22). While these *in vitro* studies typically use no-carbon conditions or chemical inhibition of tRNA synthetases to induce the stringent response, whether *E. coli* or other commensal bacteria engage these pathways during routine or extreme nutrient fluctuations in the gut is unexplored.

Commensal gut anaerobes also produce (p)ppGpp (23, 24). In the prominent gut commensal *Bacteroides thetaiotaomicron,* (p)ppGpp levels are controlled by two enzymes, BT0700 (which encodes a (p)ppGpp synthase domain) and BT3998 (which encodes both synthase and hydrolase domains) (24). As observed in *E. coli, B. thetaiotaomicron* requires (p)ppGpp to respond to environmental stresses and nutrient limitation, including oxygen exposure and carbon starvation (23, 24). Notably, a (p)ppGpp^0^ strain (that lacks BT0700 and BT3998) exhibits a loss of viability during carbon starvation and a significant fitness defect in gnotobiotic mice despite exhibiting normal exponential growth in rich and minimal medium in *vitro* (24). When and why this signal to halt growth is important in the competitive gut environment is unclear.

Here, we report that mouse gut microbial communities, and human gut microbiomes transplanted into gnotobiotic mice, respond to brief periods when the host is not eating through a transient wave of (p)ppGpp production. This adaptation is mirrored in the model human gut commensal *B. thetaiotaomicron*; in this species, (p)ppGpp^0^ strains exhibit a disrupted transcriptional program and significantly drop in population size within 12 h of host fasting. Together, these studies identify a general and conserved mechanism that allows gut microbiomes to align bacterial physiology with a time-restricted feeding regimen of their host.

## RESULTS

### *B. thetaiotaomicron* gene expression profiles vary during a timed feeding regimen in monoassociated gnotobiotic mice

**Fig 1:**
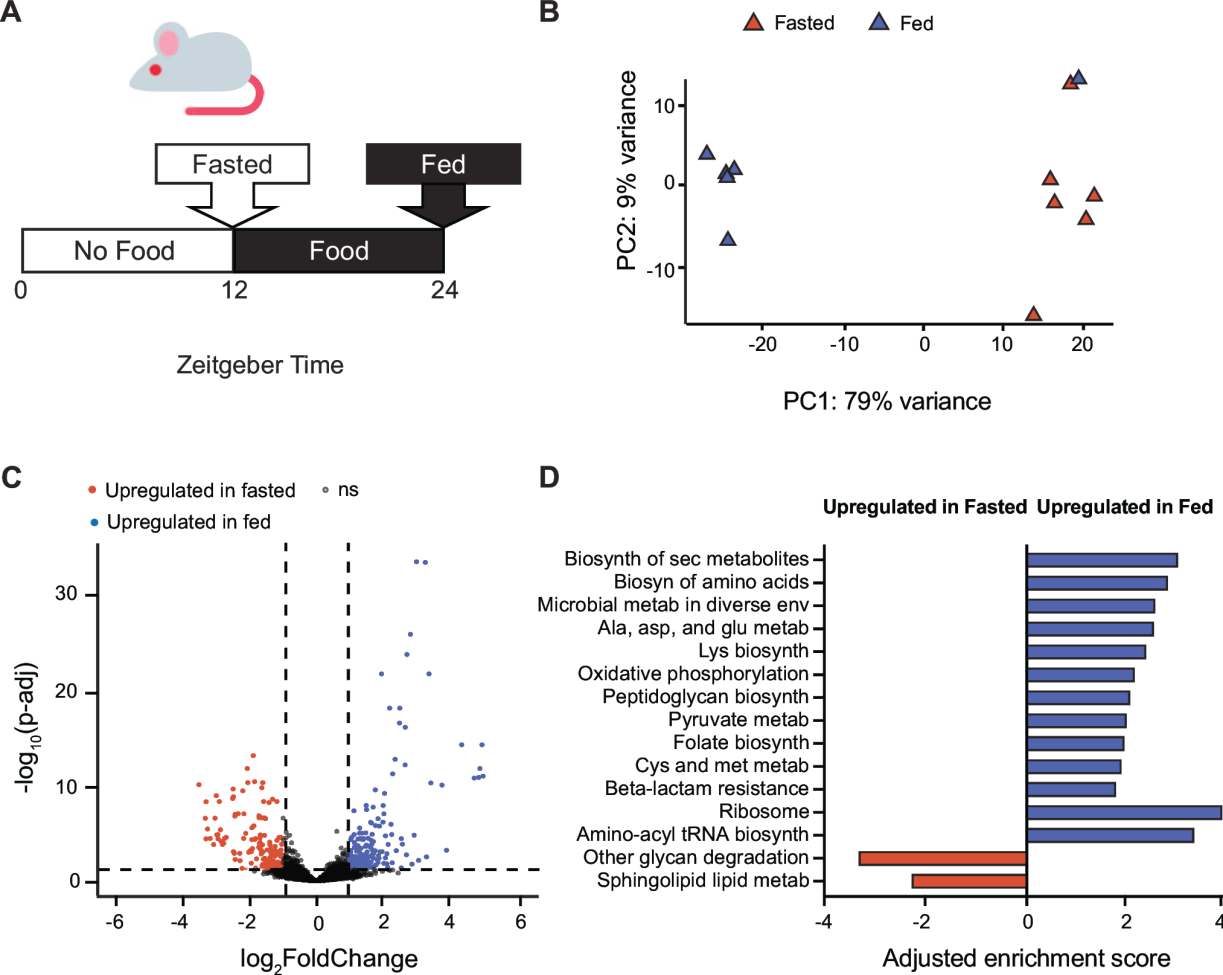
*B. thetaiotaomicron* alters its transcriptome during a time-restricted feeding regimen in monoassociated gnotobiotic mice. (**A**) Schematic of the mouse-feeding schedule. (**B**) Principal component analysis (PCA) of wild-type (WT) *B. thetaiotaomicron* transcriptomes during fed (blue) and fasted (orange) timepoints. Samples were collected from cecal contents of monoassociated gnotobiotic mice. (**C**) Volcano plot of WT *B. thetaiotaomicron* gene expression during fed and fasted timepoints. Gene expression changes were filtered for significance using a false discovery rate (FDR) of <0.05 and a log_2_fold change >1 or <−1. Log_2_fold change and *P*-adjusted value thresholds are represented by dotted lines. Genes significantly upregulated in fed or fasting phases are colored in blue or orange, respectively. (**D**) Gene set enrichment analysis of WT *B. thetaiotaomicron* gene expression during fed and fasted timepoints. Adjusted normalized enrichment scores are plotted for significant gene sets with a nominal *P*-value <0.05 and FDR <0.25. “Biosynthesis,” “secondary,” “metabolism,” and “environments” are abbreviated.

To investigate how gut microbes change their physiology to adapt to the diurnal fasting patterns of the host, we colonized germfree mice with *B. thetaiotaomicron* and maintained these animals on a periodic feeding schedule in which food was available for 12 h during the dark phase, when mice are generally active and eating, and removed for 12 hours during the light phase, when mice are generally resting and not eating (Fig. 1A). This schedule allowed us to simulate the periodic feeding that humans follow (eating during active periods, fasting during resting periods). We conducted RNA sequencing on cecal contents collected at ZT24 (at the end of the feeding period, “fed” state) and ZT12 (at the end of the fasting period, “fasted” state).

Principal component analysis (PCA) of normalized transcriptomic data showed that PC1, which explains ~80% of the observed variance, separates fed-state from fasted-state samples and suggests that these brief fasting and feeding periods affect the transcriptional profile of *B. thetaiotomicron* (Fig. 1B). Comparison of *B. thetaiotaomicron* transcriptomes between fed (ZT24) and fasted (ZT12) timepoints identified a subset of genes (348/4,902, representing ~7% of the genome) that were significantly differentially regulated between these conditions (twofold cutoff, *P* < 0.05 after multiple hypothesis testing correction) (Fig. 1C). To identify pathways upregulated in the fed or fasted state, we conducted gene set enrichment analysis (GSEA) (25) using *B. thetaiotaomicron* KEGG GO pathways (26) (Fig. 1D). Notably, aminoacyl-tRNA biosynthesis and ribosomal subunit production pathways were the most significantly enriched among the sets of genes upregulated in the fed state and repressed in the fasted state. By contrast, genes upregulated in the fasted state were enriched in glycan degradation pathways and sphingolipid metabolism, both of which include PULs responsible for metabolism of host and diet-derived polysaccharides.

**Fig 2:**
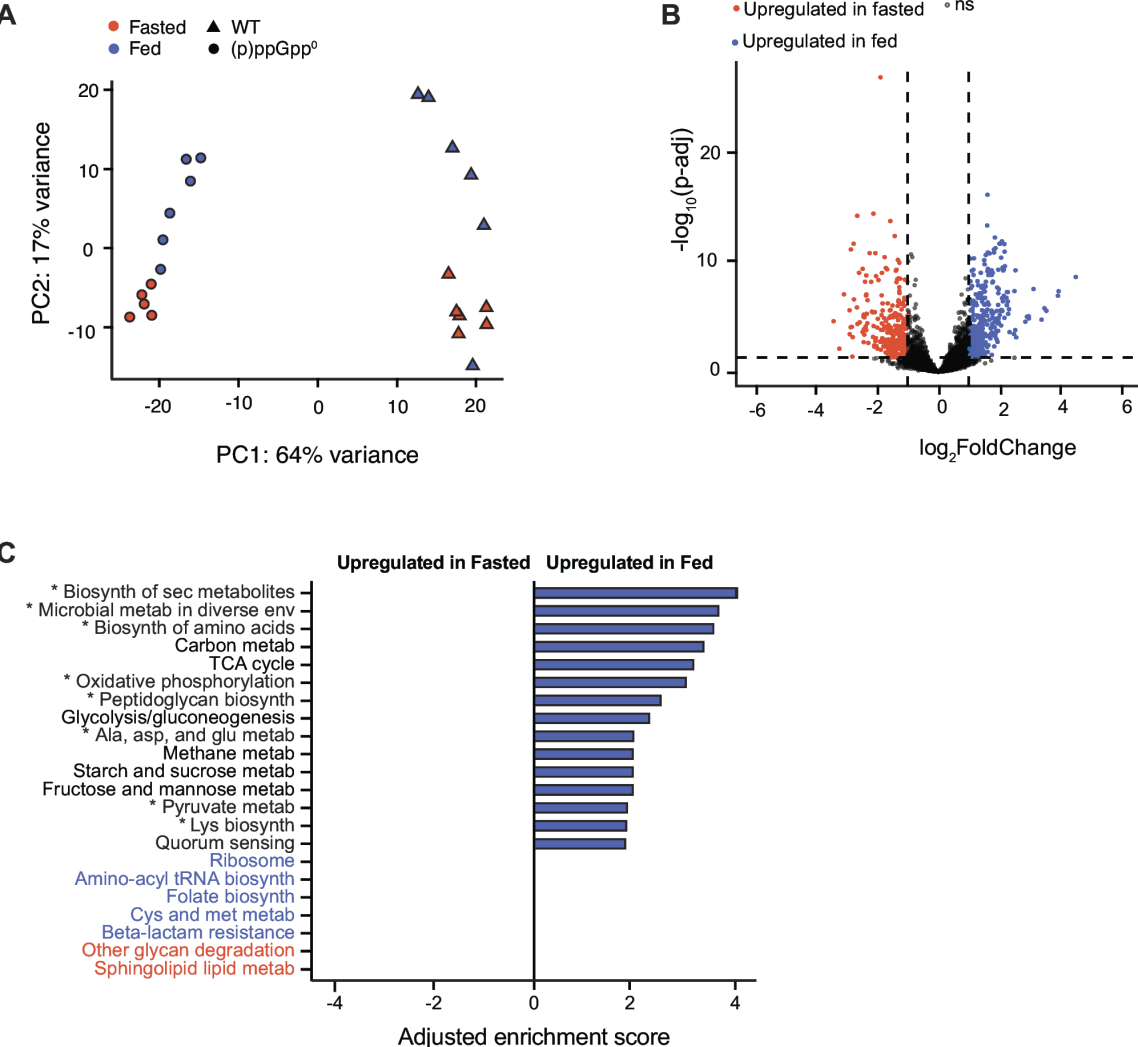
*B. thetaiotaomicron* exhibits (p)ppGpp-dependent gene expression changes during host fed and fasted phases. (**A**) Principal component analysis plot of WT (triangle) and (p)ppGpp^0^ (circle) *B. thetaiotaomicron* transcriptomes during fed (blue) and fasted (orange) timepoints. (**B**) Volcano plot of gene expression of the *B. thetaiotaomicron* (p)ppGpp^0^ strain during fed and fasted timepoints. Significantly differentially expressed genes (FDR < 0.05 and a log_2_fold change >1 or <−1) are colored in blue and orange. (**C**) Gene set enrichment analysis of *B. thetaiotaomicron* (p)ppGpp^0^ gene expression during fed and fasted timepoints. Adjusted normalized enrichment scores are plotted for significant gene sets with a nominal *P*-value <0.05 and FDR <0.25. Gene sets upregulated during the Fed state as in the WT strain are denoted with asterisks. Gene sets upregulated in the fasted or fed states in the WT but not the (p)ppGpp^0^ strain are noted in orange and blue text, respectively. “Biosynthesis,” “secondary,” “metabolism,” and “environments” are abbreviated.

Repression of aminoacyl-tRNA biosynthesis and ribosomal subunit production are both associated with the stringent response in diverse bacteria, including *B. thetaiotaomicron* (24, 27–29). We reasoned that if repression of aminoacyl-tRNA and ribosomal subunit pathways during the fasted phase was due to increased (p)ppGpp levels, a (p)ppGpp^0^ strain would fail to repress these pathways at this time point. To test this, we colonized germfree mice with a *B. thetaiotaomicron* (p)ppGpp^0^ strain (24) and conducted RNA-seq on cecal contents collected in the fed and fasted timepoints as above. PCA of wild-type (WT) and (p)ppGpp^0^ transcriptomes from these four groups showed that samples clustered first by *B. thetaiotaomicron* genotype, then by fed or fasted state (Fig. 2A), suggesting that the ability to produce (p)ppGpp affects gene expression in both states. This concurs with previous studies of gene expression in wild-type and (p)ppGpp^0^ *B. thetaiotaomicron* strains collected from monocolonized mice at an intermediate time of day (24). Compared to the wild-type strain, the (p)ppGpp^0^ strain differentially regulated nearly twice as many genes (659/4,902) between the fed and fasted time points (Fig. 2B). Many of these changes in gene expression in the (p)ppGpp^0^ strain are distinct from those found in the wild-type strain (Fig. S1A); shared responses are distributed between fed-upregulated and fasting-upregulated genes (Fig. S1B). Notably, GSEA analyses revealed that the (p)ppGpp^0^ strain failed to repress aminoacyl-tRNA biosynthesis and ribosomal subunit production pathways at the fasted time point relative to the fed timepoint, suggesting that these gene expression changes are (p)ppGpp-dependent in the wild-type strain (Fig. 2C). Together, these studies suggest that the (p)ppGpp^0^ strain exhibits a distinct transcriptional profile during both fed and fasted timepoints and that this signaling pathway is required for the stringent response signature exhibited by the wild-type strain during the fasted timepoint.

### *B. thetaiotaomicron* increases ppGpp levels during the fasting period of a timed-feeding regimen in mice

**Fig 3:**
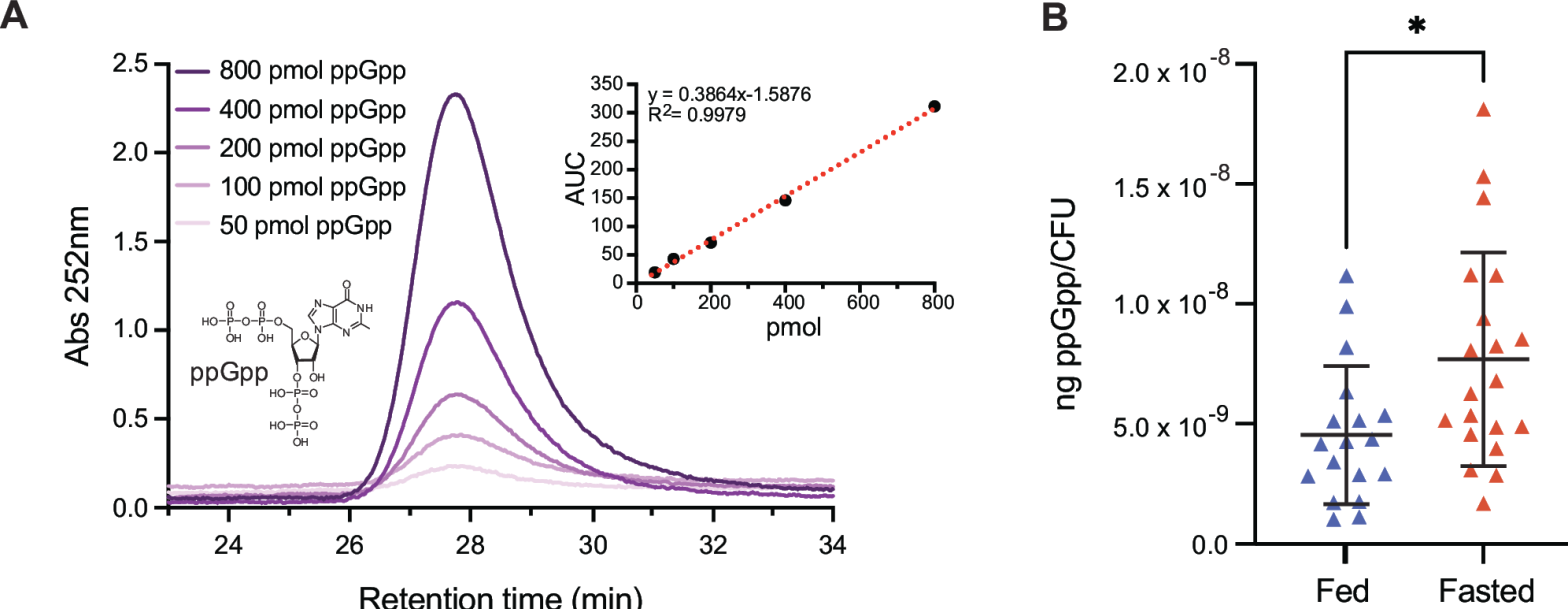
*B. thetaiotaomicron* increases ppGpp levels during the fasted phase of a time-restricted feeding regimen. (**A**) Chromatogram traces of HPLC-based ppGpp quantification. Varying shades of purple represent different concentrations of ppGpp standard. The structure of ppGpp (lower left) and area under the curve values (upper right) are also provided. (**B**) Cecal ppGpp levels during fed (blue) and fasted (orange) phases in WT *B. thetaiotaomicron* monoassociated gnotobiotic mice. Significance is indicated with asterisks (*P < 0.05, Mann-Whitney *U* test).

We next reasoned that if *B. thetaiotaomicron* alters its gene expression program in a (p)ppGpp-dependent manner between fed and fasted host states, the levels of this intracellular signal would be modulated accordingly. To test this, we first adapted and validated a ppGpp extraction and HPLC-based quantification method (30, 31) (Fig. S2A) that allowed the detection of this molecule at a lower limit of 2.5 µM (Fig. 3A; Fig. S2B). In cecal contents collected from *B. thetaiotaomicron* (p)ppGpp^0^-monoassociated gnotobiotic mice, ppGpp was not detected, establishing the specificity of the method in the presence of all other *B. thetaiotaomicron* and host metabolites in these gut contents (Fig. S2B).

We next colonized germfree mice with wild-type *B. thetaiotaomicron*, provided food as described in Fig. 1A, and measured cecal ppGpp concentrations normalized to colony forming units (CFU) at the fed and fasted phase (Fig. 3B). Notably, ppGpp levels were significantly elevated in the fasted phase relative to the fed phase, consistent with the (p)ppGpp-associated changes in gene expression during the fasted timepoint.

### *B. thetaiotaomicron* maintains constant abundance in fed and fasted mice in a (p)ppGpp-dependent manner

**Fig 4:**
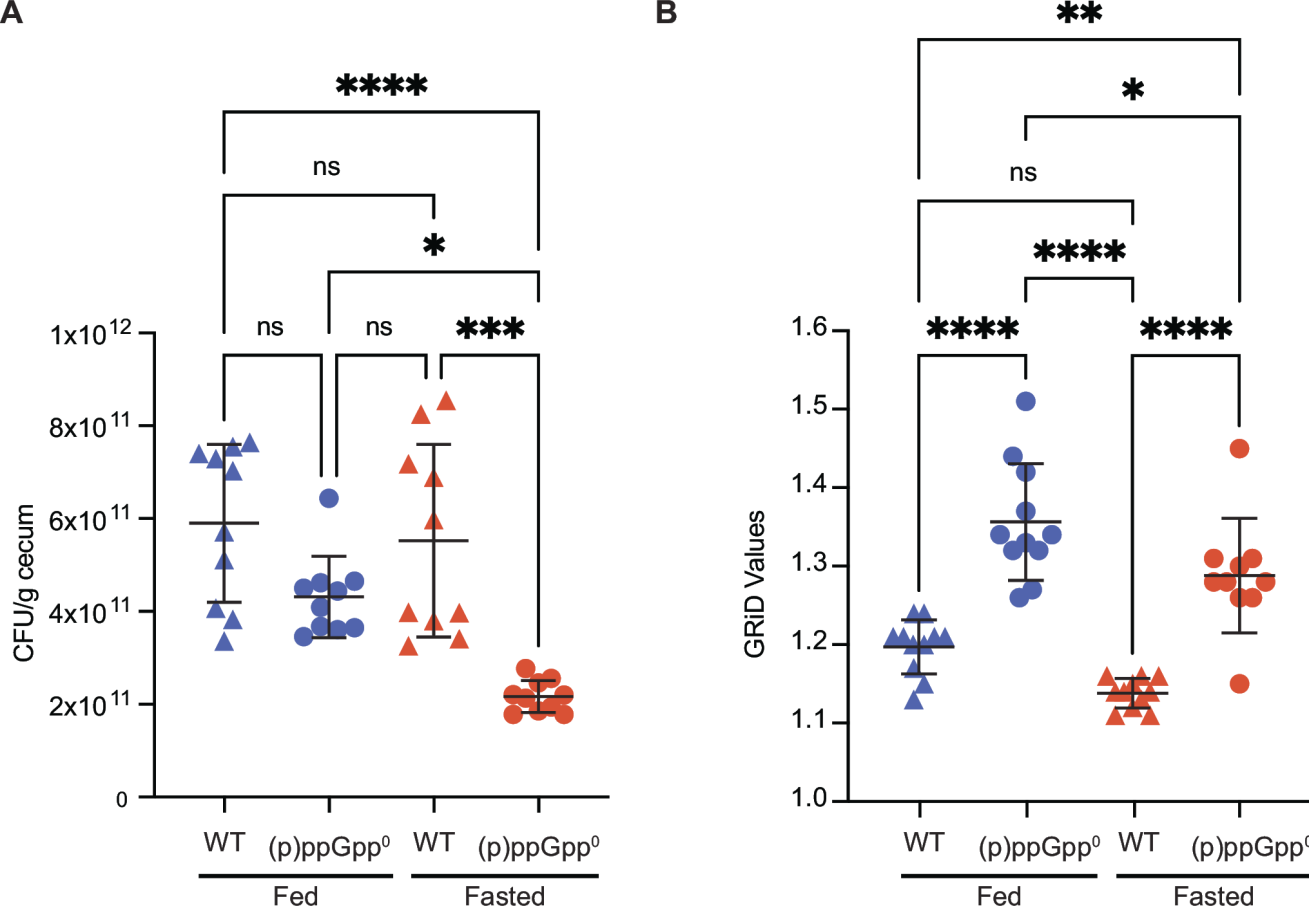
*B. thetaiotaomicron* requires (p)ppGpp to maintain abundance and coordinate chromosomal replication during fed and fasted phases. (**A**) Bacterial density (colony forming units per gram cecal content) during fed (blue) and fasted (orange) phases in gnotobiotic mice monoassociated with *B. thetaiotaomicron* WT or (p)ppGpp^0^ strains. (**B**) GRiD values during fed (blue) and fasted (orange) phases in gnotobiotic mice monoassociated with *B. thetaiotaomicron* WT or (p)ppGpp^0^ strains. GRiD values represent the peak-to-trough ratio of the number of sequencing reads mapping to the *B. thetaiotaomicron* origin and terminus of replication in each condition. In both panels, significance is indicated with asterisks (*P < 0.05; **P < 0.01; ***P < 0.001, ****P < 0.0001) based on a Kruskal-Wallace test followed by Dunn’s multiple comparison test.

*In vitro*, *B. thetaiotaomicron* uses (p)ppGpp to maintain viability during carbon starvation; the (p)ppGpp^0^ strain exhibits a significant loss of viability under these conditions (24). To test the hypothesis that (p)ppGpp production in the fasted mouse gut similarly enables *B. thetaiotaomicron* to maintain its abundance when the host is not eating, we measured bacterial abundance (as determined by CFU/g cecal contents) of *B. thetaiotaomicron* wild type and (p)ppGpp^0^-monoassociated mice in fed and fasted states. Indeed, despite the lack of food consumption by the host during the fasting period, wild-type *B. thetaiotaomicron* maintains constant abundance at the fed and fasted phases; furthermore, wild-type and (p)ppGpp^0^ strains do not significantly differ in abundance during the fed state (Fig. 4A; if the mouse with highest colonization in the (p)ppGpp^0^ fed group is removed from the analysis, there remains no significant difference between groups). By contrast, the abundance of the (p)ppGpp^0^ strain drops significantly (by 53%, *P* < 0.05) during the fasted phase compared to the fed phase; this colonization level is also significantly lower than the wild-type strain at either timepoint (Fig. 4A).

### *B. thetaiotaomicron* requires (p)ppGpp to coordinate chromosomal replication with a timed feeding regimen in mice

One of the hallmark features of (p)ppGpp production in *E. coli* and *B. subtilis* is the reduction of chromosomal replication rate, which allows bacteria to coordinate DNA replication with growth (32–34). Consequently, *E. coli* (p)ppGpp^0^ strains have higher chromosomal replication rates compared to their wild-type counterparts *in vitro*, regardless of growth rate (32, 34). We reasoned that if (p)ppGpp production modulates *B. thetaiotaomicron* chromosomal replication in the gut, wild-type bacteria would exhibit reduced chromosomal replication in the gut compared to the (p)ppGpp^0^ strain. To estimate *B. thetaiotaomicron* chromosomal replication rates, we conducted peak-to-trough analysis, which uses shotgun DNA sequencing to evaluate the relative abundance of DNA mapping to the chromosomal origin of replication compared to the terminus (35, 36). High peak-to-trough ratios reflect the initiation of one or more rounds of chromosomal replication at the origin before the previous round of replication is completed (higher replication rate), whereas lower ratios indicate fewer rounds of replication occurring concurrently (lower replication rate) (35). As expected, peak-to-trough analysis of *B. thetaiotaomicron in vitro* cultures revealed significantly higher peak-to-trough ratios in exponential phase compared to stationary phase or carbon starvation; the (p)ppGpp^0^ strain exhibited similar behavior (although with slightly but significantly higher peak-to-trough ratios during the stress conditions; Fig. S3). We colonized germfree mice with either wild-type or (p)ppGpp^0^ *B. thetaiotaomicron,* transferred animals to the restricted feeding schedule as previously described (Fig. 1A), and collected cecal contents at ZT12 and ZT24 for shotgun DNA sequencing and calculation of peak-to-trough ratios. Notably, the (p)ppGpp^0^ strain exhibited significantly elevated peak-to-trough ratios relative to the wild-type strain at both time points (Fig. 4B). These results suggest that *B. thetaiotaomicron* uses (p)ppGpp to limit chromosomal replication rates in the gut.

### *B. thetaiotaomicron* utilizes host-associated glycans in a (p)ppGpp-dependent manner

In addition to modulating chromosomal replication rate, (p)ppGpp production has widespread impacts on gene expression (Fig. 2B and C). We reasoned that in addition to (p)ppGpp-repressed genes (encoding aminoacyl-tRNAs and ribosomal subunits), genes activated during fasting in a (p)ppGpp-dependent manner could also contribute to the ability of *B. thetaiotaomicron* to maintain its abundance in the fasted phase and reveal differences in the niche of wild-type and (p)ppGpp^0^ strains when the host is not eating. GSEA analysis highlights “other glycan degradation” and “sphingolipid metabolism” as the pathways enriched among fasting-upregulated genes in the wild-type strain but not in the (p)ppGpp^0^ strain (Fig. 1D and 2C).

**Fig 5:**
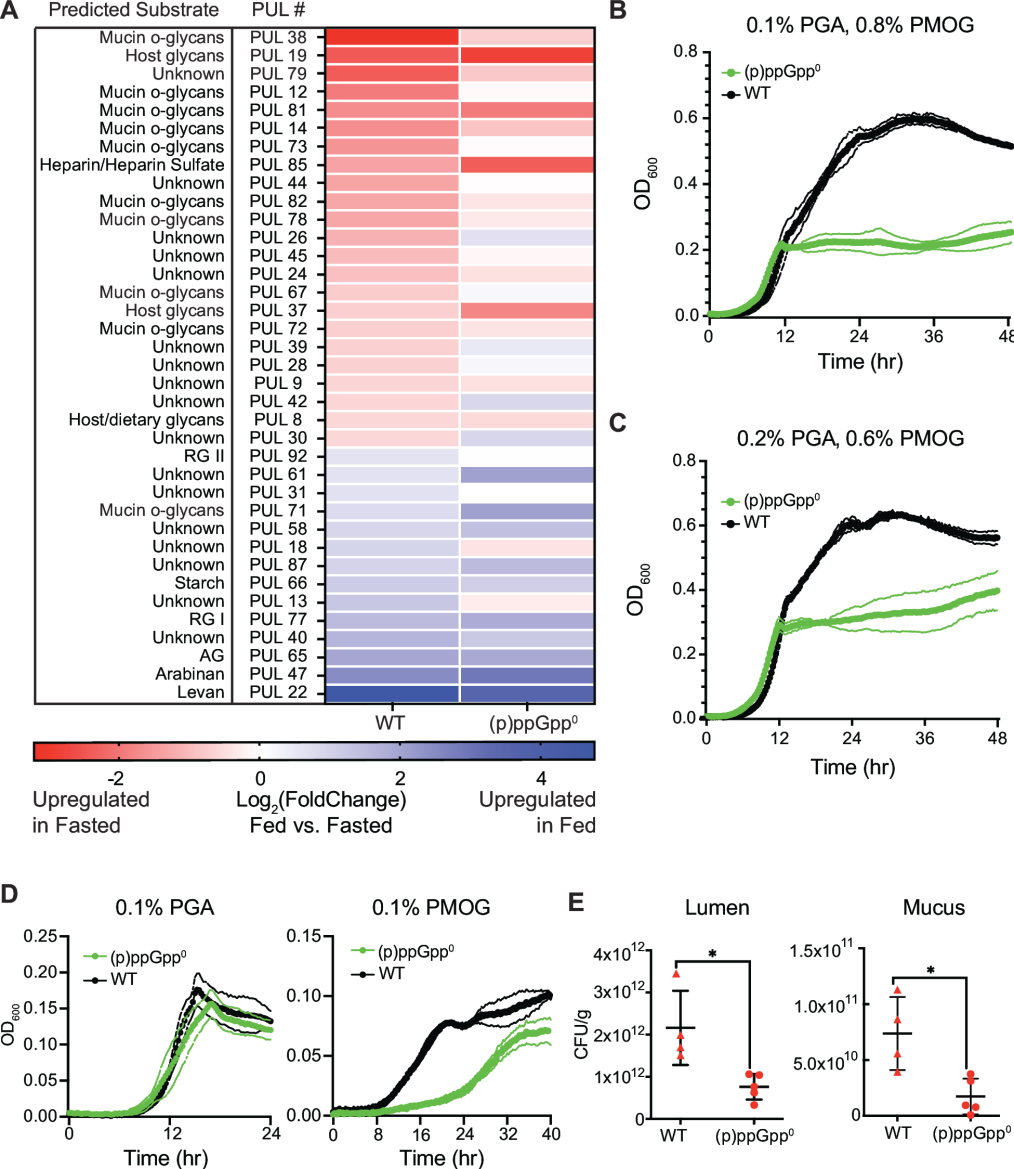
*B. thetaiotaomicron* utilizes host-associated glycans in a (p)ppGpp-dependent manner. (**A**) Heatmap of gene expression of *susC/D* pairs in *B. thetaiotaomicron* WT or (p)ppGpp^0^ strains in cecal contents of monoassociated gnotobiotic mice during fed or fasted timepoints. PUL number and predicted substrates are from PULDB (40). PULs shown are those with altered expression in the WT strain between fed and fasted mice [*susCD* log_2_(FoldChange) >0.5 or <−0.5]. Rhamnogalacturonan and arabinogalactan are abbreviated as RG and AG, respectively. PULs 14, 82, and 72 also metabolize N-glycans. (**B**) Growth of *B. thetaiotaomicron* WT (black) and (p)ppGpp^0^ (green) strains in minimal medium containing 0.1% (wt/vol) polygalacturonic acid (PGA) and 0.8% (wt/vol) porcine mucin O-glycans (PMOG) as the sole carbon source. (**C**) Growth of WT and (p)ppGpp^0^ strains in minimal medium containing 0.2% PGA and 0.6% PMOG. (**D**) Growth of WT and (p)ppGpp^0^ strains in minimal medium containing either 0.1% PGA (left) or 0.1% PMOG (right) as the sole carbon source. (**E**) Bacterial density during the fasted timepoint in cecal lumen (left) or mucus layer (right) in gnotobiotic mice monoassociated with *B. thetaiotaomicron* WT or (p)ppGpp^0^ strains. Significance is indicated with asterisks (*P < 0.05, Mann-Whitney *U* test).

Notably, most (29/38, ~75%) of the genes in these pathways are encoded in PULs. To investigate PUL regulation during the fed and fasted phases, we compared the expression of *susC* and *susD* genes between the wild-type and (p)ppGpp^0^ strains in fed and fasted states (Fig. 5A). *SusC* and *susD* are conserved across PULs, encode the central transport components of these multiprotein machines (37), and are generally expressed in response to their substrate (38). Consistent with published observations that *B. thetaiotaomicron* upregulates host glycan-targeting PULs in the presence of host glycans if plant polysaccharides are limiting (39), the wild-type strain upregulated multiple PULs predicted to target host glycans during the fasted timepoint (Fig. 5A). By contrast, the (p)ppGpp^0^ strain failed to upregulate many of these host glycan-targeting PULs during this timepoint (Fig. 5A).

In the presence of dietary and mucosal glycans *in vitro, B. thetaiotaomicron* generally prioritizes plant polysaccharides and upregulates host glycan-targeting PULs once the plant polysaccharides are depleted (39, 41). We measured growth of the wild-type strain in minimal medium containing a representative diet-derived plant polysaccharide (polygalacturonic acid; PGA) in combination with host-derived glycans (porcine mucin O-glycans; PMOG). The wild-type strain exhibits diauxic growth in this substrate combination as expected (39), with the dietary polysaccharide PGA consumed first; altering the PGA:PMOG ratio from 0.1%:0.8% to 0.2%:0.6% (wt/vol) increases growth during the first stage of exponential growth (Fig. 5B and C), and PGA-responsive PULs are upregulated during this stage (Fig. S4). Consistent with prior reports (39), PMOG-responsive PULs are upregulated during the second stage of exponential growth (Fig. S4).

These growth dynamics are profoundly altered in the (p)ppGpp^0^ strain, which grows readily during the first (PGA) exponential phase but does not exhibit a second exponential phase and instead fails to grow once PGA is likely exhausted from the culture medium (Fig. 5B and C; Fig. S5). To further verify that the growth defect of the (p)ppGpp^0^ strain under these conditions results from a lack of ability to grow on PMOG, we cultured each strain on each substrate separately (Fig. 5D). Indeed, the wild-type and (p)ppGpp^0^ strains grew similarly on PGA alone, while the latter exhibited a drastic growth defect in minimal medium containing PMOG as the sole carbon source (Fig. 5D). The wild-type and (p)ppGpp^0^ strains expressed many PGA- and PMOG-responsive PUL genes similarly in these *in vitro* conditions (one PGA-responsive and one PMOG-responsive PUL gene exhibited statistically significant differences in expression levels although both strains regulated these genes in the same direction), suggesting that the growth defect of the (p)ppGpp^0^ strain in PMOG is likely not due to transcriptional regulation of these genes (Fig. S4B).

We reasoned that if the (p)ppGpp^0^ strain cannot grow efficiently on mucin O-glycans, it would exhibit a colonization defect in the mucus layer *in vivo*. To test this, we colonized germfree mice with wild-type or (p)ppGpp^0^ *B. thetaiotaomicron*, provided food as described in Fig. 1A, and measured levels of lumen- and mucus-associated bacteria in the cecum at the fasted time point. While luminal colonization of the (p)ppGpp^0^ strain was ~twofold lower than the wild-type strain (consistent with Fig. 4A), mucosal colonization was ~fourfold lower in the (p)ppGpp^0^ strain compared to the wild-type strain (Fig. 5E), showing that this decrease in abundance during fasting is further exacerbated in the mucus layer.

### Complex bacterial communities modulate ppGpp levels during a timed host feeding regimen

**Fig 6:**
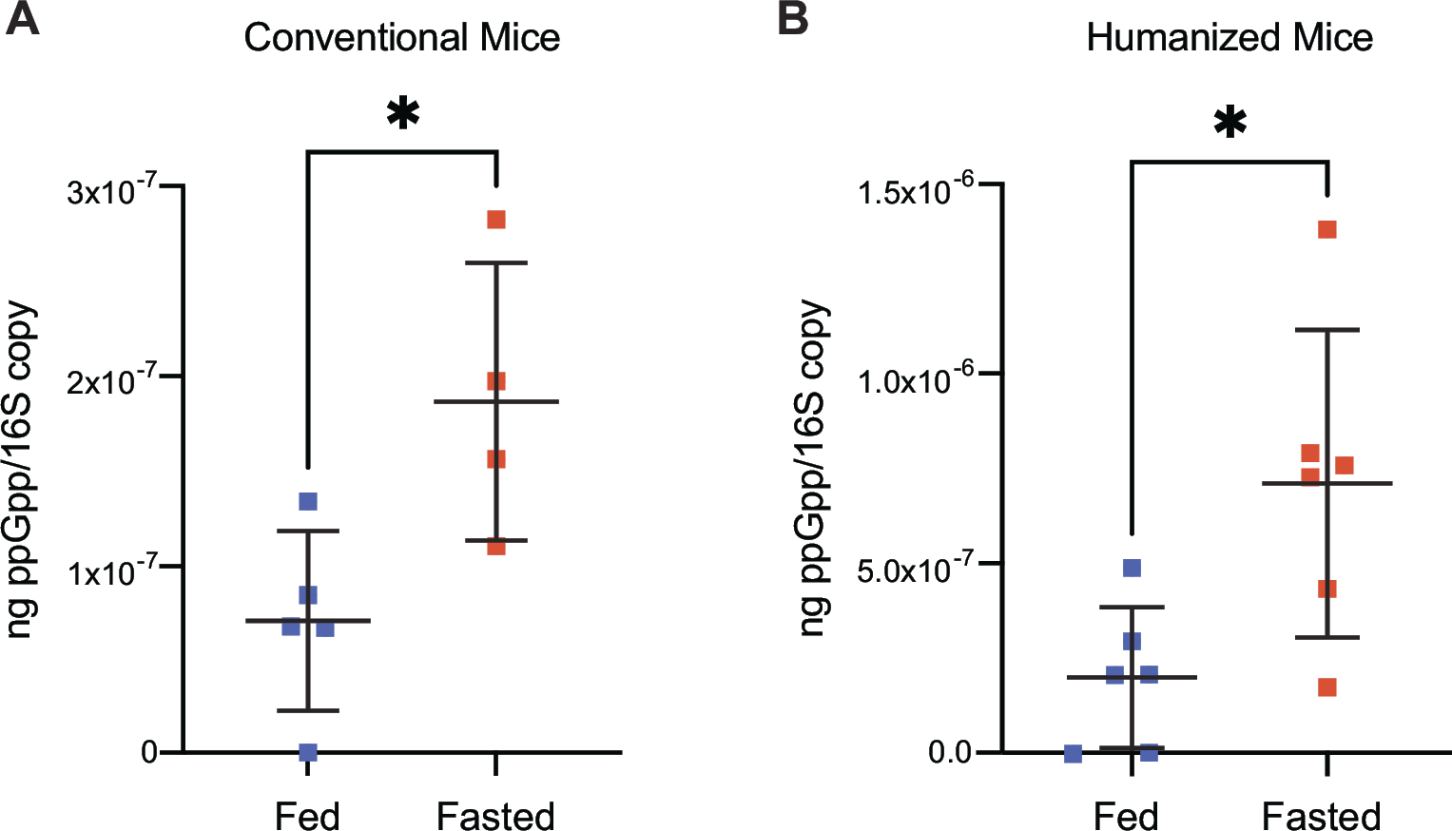
Complex microbial communities increase ppGpp levels in fasting mice. (**A**) Cecal ppGpp levels during fed (blue) and fasted (orange) phases in conventional mice carrying a complete microbiome. (**B**) Cecal ppGpp levels during fed (blue) and fasted (orange) phases in gnotobiotic mice colonized with complete human fecal microbiomes. Significance is indicated with asterisks (*P < 0.05, Mann-Whitney *U* test).

To determine if the coordination of ppGpp production with host eating also occurs in complex microbial communities, we placed conventional C57BL/6 mice on the feeding/fasting schedule described in Fig. 1A and measured cecal ppGpp concentrations at fasted and fed states as above. As observed in *B. thetaiotaomicron* monocolonized mice, these complex communities significantly elevated ppGpp levels in the fasted timepoint (Fig. 6A). Next, we repeated this experiment with gnotobiotic mice carrying human fecal microbiomes; as observed in conventional mice, these human gut communities similarly increased ppGpp levels at the fasted timepoint (Fig. 6B). Together, these results suggest that the ability of *B. thetaiotaomicron* to coordinate ppGpp production with host eating extends to other species and is conserved across mammalian gut microbiomes.

## DISCUSSION

In the gut, bacteria must compete for limited space and nutrients in a dynamic environment (3). In this report, we demonstrate that both mouse and human gut microbiomes respond to host feeding dynamics through the production of the intracellular second messenger (p)ppGpp. This response is recapitulated by the prominent gut commensal *B. thetaiotaomicron*, whose transcriptional response to short-term host fasting highlights genes associated with the stringent response. *B. thetaiotaomicron* requires (p)ppGpp to correctly regulate chromosomal replication, maintain viable cell density through the feeding-fasting cycle, and utilize host-associated glycans when the host is not eating.

Quantification of (p)ppGpp in complex communities and environments such as the gut is challenging due to the inherent instability of these compounds. Measurement of expression of (p)ppGpp-dependent genes (42) can provide an indirect indication of (p)ppGpp levels, although these genes are often regulated by multiple signals (43–45). Thin-layer chromatography (TLC) (46), (p)ppGpp-responsive biosensors (47), and mass spectrometry (30) provide direct approaches for (p)ppGpp quantification. However, the requirement for radiolabeled precursors (for TLC), genetic tractability and suitable reporters (for biosensors), or specialized equipment (for mass spectrometry) complicate the application of these strategies to host-associated environments or natural microbial communities. Here, we adapt an HPLC-based strategy (30, 31) that can be readily applied to individual species and complex communities in the mammalian gut (and potentially other) environments.

The specific signals that trigger ppGpp production during the fasting phase of a timed host feeding regimen in *B. thetaiotaomicron* and human and mouse gut microbial communities are unknown. Mice generally consume food at night and fast during the day, and it is possible that nutritive (fasting), visual (daytime), and/or circadian cues contribute to the (p)ppGpp response in the gut microbiome. Carbon starvation directly activates (p)ppGpp production in *B. thetaiotaomicron* and many other bacteria (16, 24, 48); fasting-dependent changes in host physiology, including altered hormones or gut motility, could also affect (p)ppGpp production (49). Furthermore, diurnal feeding establishes oscillations in antimicrobial peptide and MHC Class II expression in the small intestine and IgA secretion in the colon of mice (12, 50–52), which could also provide (p)ppGpp-inducing signals to gut commensal bacteria. Because food consumption is the primary driver of day/night differences in gut antimicrobial peptide production (12, 53) and microbiome community composition (8, 10), we expect that the major signals governing (p)ppGpp production also derive from food consumption. While a day-restricted feeding schedule could potentially be used to further explore these signals, this stressful condition induces arrhythmicity in gene expression and metabolic syndrome-like pathology in mice (54–58), potentially complicating the interpretation of (p)ppGpp measurements in the gut of day-fed mice. Given the global nature of (p)ppGpp signaling in bacteria, it is also likely that this response plays additional roles in the gut environment during both fed and fasting states.

Although (p)ppGpp induction alters gene expression in many bacteria (20–22, 24), wild-type *B. thetaiotaomicron* changes the expression of far fewer genes between the fed and fasted state (348) compared to the (p)ppGpp^0^ strain (659) (Fig. 1C vs Fig. 2B; Fig. S1). Additionally, these strains alter gene expression between fasted and fed states in different ways: of the 659 differentially expressed genes in the (p)ppGpp^0^ strain, only 101 (~15%) are similarly regulated in the wild-type strain (Fig. S1A). In *E. coli,* isoleucine starvation *in vitro* (which triggers (p)ppGpp production in this species) alters the expression of significantly fewer genes in a wild-type strain compared to a (p)ppGpp^0^ strain, with only 20% similarly regulated in both strains (20). In the gut environment, the increased number of differently expressed genes in the *B. thetaiotaomicron* (p)ppGpp^0^ strain likely reflects compensatory gene regulation in the absence of (p)ppGpp-mediated physiological adaptation. The (p)ppGpp^0^ strain cannot maintain wild-type colonization density during fasting (Fig. 4A), suggesting that these compensatory measures are insufficient to replace the function of (p)ppGpp signaling during host fasting. It should be noted that the wild-type and (p)ppGpp^0^ strains exhibit many transcriptional differences in both the fed and fasted states, and it is likely that (p)ppGpp signaling is required for competitive fitness in both conditions (24).

Notably, *B. thetaiotaomicron* upregulates gene sets involved in glycan and sphingolipid metabolism in a (p)ppGpp-dependent manner during host fasting (Fig. 1D and 2C). Many of these genes are localized in PULs, and many predicted mucin O-glycan targeting *susC/susD* pairs are upregulated in the fasted gut by wild-type but not (p)ppGpp^0^ *B. thetaiotaomicron* (Fig. 5A). *In vitro*, the (p)ppGpp^0^ strain induces expression of representative PMOG-responsive PULs during a shift from PGA to PMOG, yet fails to grow on PMOG (Fig. 5B and D). This suggests that expression of other genes could be required for growth on PMOG or that (p)ppGpp influences the expression of PMOG utilization machinery at the post-transcriptional level. Indeed, (p)ppGpp directly binds and modulates the function of multiple enzymes across diverse bacterial phyla (28, 59–62).

Given that the (p)ppGpp^0^ strain has the capacity to upregulate representative mucin O-glycan-responsive PULs when exposed to PMOG *in vitro,* why does this strain fail to induce these genes *in vivo*? One possibility is that the fraction of the *B. thetaiotaomicron* population exposed to the inducing signals in the fasted gut is lower in the (p)ppGpp^0^ strain compared to the wild type. Indeed, direct CFU measurements suggest that the mucus-associated population in *B. thetaiotaomicron* (p)ppGpp^0^-monoassociated mice is reduced by ~75% compared to the mucus-associated population in *B. thetaiotaomicron* wild-type-monoassociated mice (Fig. 5E). Alternatively, murine O-glycans could provide different (p)ppGpp-dependent signals compared to PMOG, although the wild-type strain induces predicted mucin O-glycan-responsive PULs both during the fasted state in mice and in response to PMOG *in vitro* (Fig. 5A; Fig. S4B). Taken together, these results suggest that *B. thetaiotaomicron* could use (p)ppGpp signaling to orchestrate the utilization of multiple carbon sources in the gut. *E. coli* (p)ppGpp^0^ strains exhibit limited metabolism of a variety of carbon sources *in vitro* (20), indicating that these connections could extend to other gut microbes.

Most gut bacteria possess enzymes to produce and degrade (p)ppGpp (16, 17). Some bacterial species, including *B. thetaiotaomicron* and *E. coli,* have a bifunctional synthase/hydrolase enzyme and a monofunctional synthase enzyme, the latter of which results from gene duplication and loss of function in the hydrolase domain (17, 24). Other bacteria, including many Firmicutes, encode a single bifunctional synthase/hydrolase with or without other small alarmone synthases, which contain only a (p)ppGpp synthase domain (17). The diverse repertoire of (p)ppGpp synthase and hydrolase enzymes across members of the gut microbiome suggests that the signals that regulate (p)ppGpp levels, and the functions under (p)ppGpp control, likely also vary across these taxa. For example, in *E. coli*, the monofunctional synthase (RelA) is involved in sensing amino acid starvation through direct association with the ribosome (63, 64), while the bifunctional synthase/hydrolase (SpoT) is involved in sensing fatty acid starvation through acyl carrier protein (65, 66) and carbon starvation through Ytfk (48, 67). However, the observation that mouse and human gut communities comprising hundreds of species coordinate ppGpp levels with a time-restricted feeding regimen in mice (Fig. 6A and B) suggests that this condition produces a widespread (p)ppGpp response. This could result from the coordinated activity of most members of these communities or from a subset that respond to these conditions via (p)ppGpp production. Mouse and human communities have limited species overlap (1), favoring the possibility that modulating (p)ppGpp levels under these conditions is a widespread response across community members that is conserved across the gut microbiomes of mammals.

Our results complement previous studies that have examined the role of (p)ppGpp in bacterial pathogens during infection. In these pathogens, (p)ppGpp^0^ mutants generally exhibit reduced virulence and antibiotic tolerance (19, 68–70). While our study focused on commensal members of the microbiome, the same (p)ppGpp-dependent coordination of bacterial physiology with host conditions could also play a role in pathogen colonization. Determining how microbes modulate their physiology in response to host feeding can potentially inform therapeutic approaches to various diseases. For example, insulin resistance (71, 72), drug response, and infection outcome (13, 73, 74) are each impacted by time of day and time of feeding and have also been linked to the gut microbiota. Thus, further understanding of how gut microbes coordinate their physiology with host feeding and other behaviors could reveal new factors that extend our understanding of the role of the microbiome in health and disease.

## MATERIALS AND METHODS

### Experimental model and subject details

#### Animal models

Germfree C57BL/6 mice (Taconic) were bred and maintained in the Goodman lab gnotobiotic facility on an autoclaved diet (Purina, #5K67). Mice were maintained in flexible plastic gnotobiotic isolators or sterile isocages (Allentown LLC) on a 12-h light/dark cycle. Animals were housed alone or in same-sex groups. Feeding schedules were *ad libitum* or restricted (described below). Germfree mice were colonized at 12–21 weeks of age by introduction of bacteria via gastric gavage. Within experiments, mice were mixed sex and age-matched within 2 weeks.

#### Restricted feeding

For restricted feeding experiments, mice were provided no access to food from 8:00 (denoted as ZT0) to 20:00 (denoted as ZT12) and *ad libitum* access to food from 20:00 to 8:00 (ZT12-ZT24). Room lighting was coordinated to the fasting period (lights on 8:00 to 20:00 [ZT0-ZT12]; lights off 20:00 to 8:00 [ZT12-ZT24]). At the initiation of each fasting period, bedding was replaced and enrichment removed to prevent consumption by mice; enrichment was returned at the initiation of each feeding period. Restricted feeding was maintained for 4 days before mice were sacrificed at ZT12 or ZT24, and ~100 mg aliquots of cecal material were collected into cryotubes and either immediately snap frozen in liquid nitrogen before storing at −80°C or used directly for CFU plating. Aerobic exposure was minimized during sample collection and was matched between ZT12 and ZT24 timepoints.

For *B. thetaiotaomicron* monoassociation experiments, germfree mice were colonized during the fasting phase of the first day of restricted feeding with 200 µL of stationary phase cultures (~2 × 10^7^ CFU). For colonization with human fecal samples, germfree mice were colonized 7 days before the first day of restricted feeding with 100 µL of cryopreserved human fecal samples from three separate donors (75). The same number of mice colonized with each donor microbiota was measured at each timepoint. For both humanized and conventional C57BL/6 mice (Taconic Biosciences), mice were acclimatized to isocages for 7 days before the first day of restricted feeding.

All mouse experiments were performed using protocols approved by the Yale University Institutional Animal Care and Use Committee.

#### Bacterial culture conditions

*Bacteroides thetaiotaomicron* VPI-5482 *tdk* (76), designated as the WT strain, and an isogenic ppGpp^0^ strain (either previously constructed (24) or independently generated as described below) were cultured anaerobically in glucose minimal medium [0.5% glucose, 100 mM KH_2_PO_4_ (pH 7.2), 15 mM NaCl, 8.5 mM (NH4)_2_SO_4_, 0.5 g L^−1^ cysteine, 0.2 mM histidine, 1.9 µM hematin, 100 mM MgCl_2_, 1.4 mM FeSO4, 50 mM CaCl_2_, 1 mg mL^−1^ vitamin K3, and 5 ng mL^−1^ vitamin B_12_] (14), liquid TYG medium [1% tryptone peptone (IBI Scientific), 0.5% yeast extract (Difco), 0.2% glucose, 100 mM potassium phosphate buffer pH 7.2, 0.08 mM MgSO_4_*7H_2_O, 5 mM NHCO_3_, 1.5 mM NaCl, 0.2 mM histidine, 1.9 µM hematin, 1.4 mM FeSO_4_, 50 mM CaCl_2_, 1 mg mL^−1^ vitamin K3], or on brain heart infusion (BHI) (Becton Dickinson) agar with 10% horse blood (Quad Five). Anaerobic procedures were performed in an anaerobic chamber (Coy Laboratory Products) containing 20% CO_2_, 10% H_2_, and 70% N_2_.

#### *Generation of (p)ppGpp*^*0*^ *strains*

Four *B. thetaiotaomicron* (p)ppGpp^0^ strains (with nonpolar deletions of BT0700 and BT3998) were independently constructed as described (24). Briefly, pExchange_0700 (24) was transferred into *B. thetaiotaomicron tdk* by conjugation, and second recombination events selected by plating on BHI blood agar containing 200 µg/mL 5-fluoro-2′-deoxyuridine (FUdR). After PCR verification, pExchange_3998 was used to delete BT3998 as above.

### Method details

#### RNA sample preparation

For *in vivo* RNA-seq measurements, aliquots of ~100 mg of frozen cecal samples were thawed in 10 mL of a 2:1 Bacterial RNAProtect (Qiagen):RNase-free deionized water solution, homogenized by vortexing, incubated at room temperature (RT) for 5 min, vortexed again, and pelleted by centrifugation for 10 min at 7,000*g* at 4°C. RNA was then extracted using a PowerMicrobiome kit (Qiagen) with on-column DNase treatment. After elution from the PowerMicrobiome kit, RNA was treated with TURBO DNase (ThermoFisher) in a total volume of 100 µL at room temperature for 30 min. RNA was then purified using an RNeasy kit (Qiagen) with an on-column DNase treatment (Qiagen) before being frozen at −80°C for use in RNA-seq. RNA quality was assessed using 2100 Bioanalyzer (Agilent).

For *in vitro* quantitative reverse transcription (qRT-PCR) preparation, a culture corresponding to ~5 × 10^8^ cells, or 1 mL at OD_600_ = 1, was centrifuged at 7,000 G for 5 min, decanted aerobically, quickly resuspended in 5 mL of Bacterial RNAProtect (Qiagen), and centrifuged at 7,000*g* for 5 min. Cultures were then decanted, frozen on dry ice, and transferred to −80°C until RNA extraction. Cell pellets were then thawed on ice, resuspended in 200 µL lysis buffer (10 mM Tris, 1 mM ETDA, 5 mg mL^−1^ lysozyme [Sigma], 12.5 µg mL^−1^ proteinase K [pH 8.0]), and incubated RT for 10 min. Crude RNA was extracted using an RNeasy Mini kit (Qiagen), treated with TURBO DNase (ThermoFisher) in a total volume of 100 µL at room temperature for 30 min, and processed again through an RNeasy Mini kit. Quality was assessed via gel electrophoresis before cDNA synthesis using Superscript II Reverse Trancriptase (Invitrogen) according to manufacturer’s instructions.

### Preparation and sequencing of mRNA libraries

Library preparation and RNA sequencing were performed at the Yale Center for Genome Analysis. rRNA was depleted using NEBNext rRNA Depletion Kit for bacteria (NEB), and RNAseq libraries were prepared using NEBNext Ultra II Directional RNA Library Prep Kit for Illumina (NEB), as recommended by the manufacturer. Sequencing was performed on a NovaSeq6000 S4 flow cell with 1 × 75 bp single-end sequencing to generate ~25–35 million reads per sample.

### RNA-seq analysis

Sequences were aligned to the *B. thetaiotaomicron* VPI-5482 reference genome (NC_004663) using Bowtie2 (77). Reads were counted using featureCounts (78), and differential expression analysis was carried out using DESeq2 (79). PCA plots were generated with DEseq2 data and ggplot (80). Gene set enrichment analysis was performed with GSEA (25), and volcano plots were generated with EnhancedVolcano (81). RNA-seq data are available in NCBI as BioProject PRJNA935381 (see also Table S1).

### ppGpp extraction and measurement

ppGpp was extracted as described (30) with modifications. To this end, aliquots of ~100 mg of frozen cecal samples were transferred from −80°C storage to dry ice. Samples were extracted individually by the addition of 1 mL of ice-cold 2 M formic acid along with ~250 mg of acid-washed beads (BioSpec). Samples were then transferred to ice to thaw for 5 min before disruption by bead beating (Biospec) for 2 × 20 s with 1 min on ice between each 20-s treatment. Homogenized samples were then transferred to 15-mL conical tubes (Corning), and 2 mL of ice-cold 2 M formic acid was added to each sample. After 30 min incubation on ice, 50 mM NH_4_OAc (pH 4.5) was added to each sample to bring the total volume to 6 mL. One sample per tissue group/time was split into two 3-mL aliquots in 15-mL conical tubes: 20 µL of 200 µm ppGpp or pppGpp standard (Jena Biosciences) was added to one of the aliquots to estimate extraction efficiency. Samples were then centrifuged at 2,400*g* for 5 min at 4°C or until the material was firmly pelleted at the bottom of the conical tube. Samples were then placed on ice prior to extraction using an Oasis WAX 1 cc Vac Cartridge, 30 mg Sorbent per Cartridge, 60 µm (Waters).

The Oasis WAX 1 cc Vac Cartridge was pre-treated with 1 mL of MeOH followed by 1 mL of 50 mM NH_4_OAc (pH 4.5), and the supernatant of the sample solution was loaded 1 mL at a time onto the column; the column was washed with 1 mL of 50 mM NH_4_OAc (pH 4.5), followed by 1 mL of MeOH. The nucleotide pool was eluted from the cartridge with 1 mL MeOH/H_2_O/NH_4_OH (20:70:10) solution into a 15-mL conical tube (Corning) and immediately frozen on dry ice before storing at −80°C for at least 2 h before preparing samples for lyophilization.

To lyophilize the extracted samples, tubes were transferred to dry ice, and caps were removed and replaced with a Kimwipe (KimTech) secured with a rubber band around the neck of the tube. Samples were returned to −80°C for 30 min to ensure the samples were frozen before lyophilization. Frozen samples were quickly transferred on dry ice to a lyophilizer (Labconco), and dry ice was packed around the glass holder to ensure samples remained frozen while reaching pressure. Lyophilized samples were stored at −80°C.

For measurement, lyophilized samples were dissolved in 100 µL of nuclease-free water, filtered through a Qiaquick spin column (Qiagen) by centrifugation for 1 min at 13,000*g*, and immediately used for ppGpp quantification. After measurement, samples were stored in HPLC vials at −80°C and retained similar ppGpp measurements for at least 3 months.

Chromatography was performed as described (31) with modifications. Specifically, 20 µL of extracted samples and standards were analyzed with an Agilent 1200 Infinity Series HPLC System (equipped with a Sphereclone SAX column, 4.6 × 150 mm, 5 µm) via DAD detection. The mobile phase employed a 2 mL/min isocratic delivery of 0.36 M NH_4_H_2_PO_4_ (pH 3.4, 2.5% [vol/vol] acetonitrile) up to 1 h. The detection wavelength was set to 252 nm. The mobile phase was prepared with analytical grade NH_4_H_2_PO_4_ (>99% pure), H_2_O filtered through a Milli-Q system, and HPLC-grade acetonitrile. The pH of the solution was adjusted to 3.4 through the addition of H_3_PO_4_ (85% aq. solution). Output data were converted into concentrations using a standard curve of ppGpp (Jena Biosciences).

### CFU calculations and measurements

*B. thetaiotaomicron* abundance was monitored by CFU counting on BHI-HK plates under anaerobic conditions. For groups of two, significant differences were determined by Mann-Whitney *U* test with Benjamini-Hochberg correction; for groups greater than two, significant differences were determined by Kruskall-Wallis followed by Dunn’s multiple correction.

### gDNA extraction

For gDNA extraction, ~100 mg of frozen cecal material was added to 500 µL of buffer PB (Qiagen), ~250 µL of acid-washed silica beads, 250 µL of 20% SDS, and 550 µL of phenol:chloroform pH 8.0 premixed with isoamyl alcohol (25:24:1) (VWR) in a 2-mL cryotube at room temperature. Samples were disrupted for 2 min in a Bead Beater (BioSpec), rested on ice for 1 min, and then disrupted again for 2 min. Samples were centrifuged at 8,000*g* at 4°C for 5 min, and the aqueous layer was extracted and placed on a Qiaquick spin column, centrifuged for 1 min at 13,000*g*, and then washed twice with 750 µL of PE buffer (Qiagen) before eluting in 100 µL of nuclease-free water (Qiagen).

### Whole-genome sequencing library preparation and analysis

Libraries were prepared with the IDT Lotus kit (Integrated DNA Technologies), followed by 6 cycles of amplification, and sequenced on NovaSeq6000 S4 flow cell at 40× coverage with ~15 million reads per genome. Peak-to-trough analysis was performed on WGS sequencing data using GRiD (82) package in R, using annotation from the NCBI taxonomy RefID 226186.

### PUL annotation and analyses

PUL numbers in Fig. 5A; Fig. S4B correspond to “literature-derived PULs” from the PULDB database (40). To analyze PUL gene regulation, *susC/susD* gene pairs were identified using PULDB (40) and changes in expression were calculated as the average log2(fold change) between fed and fasted phases.

### *B. thetaiotaomicron* growth measurements

*B. thetaiotaomicron* cultures were cultured for 16–20 h in minimal medium with glucose (5 mg/mL), washed twice with 2× minimal medium with no carbon source, back-diluted 1:1,000 (single carbon source experiments) or 1:200 (diauxic shift experiments) in 2× minimal medium without glucose, and aliquoted into 96-well plates (Corning). PMOG was generously provided by E. Martens and N. Pudlo (University of Michigan) and extracted from porcine mucosal glycans (Sigma Aldrich) as described (39). PMOG and PGA (Sigma Aldrich) were used at varying concentrations as indicated. Growth curves were carried out in a microplate reader (BioTek) set at 37°C and housed in an anaerobic chamber. OD_600_ measurements were collected every 15 min with shaking before each measurement. Growth measurements were conducted using both the previously constructed strain (24) and independently generated (p)ppGpp^0^ strains, with similar results.

### Quantitative reverse transcription PCR (qRT-PCR)

qRT-PCR was performed as previously described (24), using a CFX96 Realtime system (Biorad) and SYBR FAST universal qPCR master mix (KAPA Biosystems). Relative changes were calculated with ∆∆Cq method, normalizing to the xylanase gene of *B. thetaiotaomicron*. Primers are described in Table S2.

### 16S copy quantification

Femto Bacterial DNA Quantification Kit (Zymo) was used for absolute quantification of 16S gene copies in cecal samples. DNA was extracted from cecal samples as described above, and quantification of 16S gene copies was performed according to the manufacturer’s instructions (Zymo). qPCRs were prepared in 96-well plate format and performed on a CFX96 Realtime system (Biorad) with the following conditions: 10 min at 95°C, followed by 40 cycles of 30 s at 95°C, 30 s at 50°C, and 1 min at 72°C, then final extension of 7 min at 72°C. 16S concentrations were determined using a calibration curve of known concentrations of *E. coli* DNA.

### Quantification and statistical analysis

Statistical analysis of results was performed using Prism 9 (GraphPad). All graphs are expressed as means ± SEM, and *P*-values less than 0.05 were regarded as statistically significant. The statistical difference between any two groups was determined Mann-Whitney *U* test or unpaired *t*-test. For multiple groups, the statistical difference was determined using either Kruskal-Wallace test with Dunn correction or the one-way analysis of variance (ANOVA) with post-hoc Tukey test, depending on assumptions of variance.

## Data Availability

RNA-seq data have been deposited in NCBI as BioProject PRJNA935381. This paper does not report original code. Any additional information required to reanalyze the data reported in this paper is available from the corresponding author upon request.

## SUPPLEMENTAL MATERIAL

Fig. S1 mbio.01907-23-s0001.eps Limited overlap between gene expression changes in *B. thetaiotaomicron* WT and (p)ppGpp^0^ strains during fed and fasted phases in mice.

Fig. S2 mbio.01907-23-s0002.eps Representative chromatograms used in ppGpp quantification.

Fig. S3 mbio.01907-23-s0003.eps *B. thetaiotaomicron* GRiD values in varying growth conditions *in vitro*.

Fig. S4 mbio.01907-23-s0004.eps Transcriptional responses of *B. thetaiotaomicron* WT and (p)ppGpp^0^ strains during diauxic growth in PGA and PMOG.

Fig. S5 mbio.01907-23-s0005.eps Doubling time of independently constructed *B. thetaiotaomicron* (p)ppGpp^0^ strains in minimal medium containing PGA and PMOG (0.2%:0.6%).

Supplemental Legends mbio.01907-23-s0006.docx Legends for supplemental figures and tables.

Table S1 mbio.01907-23-s0007.xlsx RPKM values of (p)ppGpp^0^ and WT strains isolated from cecal contents of fed and fasted mice.

Table S2 mbio.01907-23-s0008.xlsx Primers used for qRT-PCR.

ASM does not own the copyrights to Supplemental Material that may be linked to, or accessed through, an article. The authors have granted ASM a non-exclusive, world-wide license to publish the Supplemental Material files. Please contact the corresponding author directly for reuse.

## References

[1] Ley RE, Turnbaugh PJ, Klein S, Gordon JI. 2006. Microbial ecology: human gut microbes associated with obesity. Nature 444:1022–1023. doi:10.1038/4441022a 17183309

[2] Schlomann BH, Parthasarathy R. 2019. Timescales of gut microbiome dynamics. Curr Opin Microbiol 50:56–63. doi:10.1016/j.mib.2019.09.011 31689582 PMC6899164

[3] García-Bayona L, Comstock LE. 2018. Bacterial antagonism in host-associated microbial communities. Science 361:eaat2456. doi:10.1126/science.aat2456 30237322

[4] Jensen TL, Kiersgaard MK, Sørensen DB, Mikkelsen LF. 2013. Fasting of mice: a review. Lab Anim 47:225–240. doi:10.1177/0023677213501659 24025567

[5] Kant AK. 2018. Eating patterns of US adults: meals, snacks, and time of eating. Physiol Behav 193:270–278. doi:10.1016/j.physbeh.2018.03.022 29574043

[6] Sudo N, Sekiyama M, Ohtsuka R, Maharjan M. 2009. Gender differences in "luxury food intake" owing to temporal distribution of eating occasions among adults of Hindu communities in lowland Nepal. Asia Pac J Clin Nutr 18:441–446. 19786393

[7] Huseinovic E, Winkvist A, Slimani N, Park MK, Freisling H, Boeing H, Buckland G, Schwingshackl L, Weiderpass E, Rostgaard-Hansen AL, et al.. 2016. Meal patterns across ten European countries - results from the European prospective investigation into cancer and nutrition (EPIC) calibration study. Public Health Nutr 19:2769–2780. doi:10.1017/S1368980016001142 27194183 PMC10271196

[8] Thaiss CA, Zeevi D, Levy M, Zilberman-Schapira G, Suez J, Tengeler AC, Abramson L, Katz MN, Korem T, Zmora N, Kuperman Y, Biton I, Gilad S, Harmelin A, Shapiro H, Halpern Z, Segal E, Elinav E. 2014. Transkingdom control of microbiota diurnal oscillations promotes metabolic homeostasis. Cell 159:514–529. doi:10.1016/j.cell.2014.09.048 25417104

[9] Zarrinpar A, Chaix A, Yooseph S, Panda S. 2014. Diet and feeding pattern affect the diurnal dynamics of the gut microbiome. Cell Metab 20:1006–1017. doi:10.1016/j.cmet.2014.11.008 25470548 PMC4255146

[10] Thaiss CA, Levy M, Korem T, Dohnalová L, Shapiro H, Jaitin DA, David E, Winter DR, Gury-BenAri M, Tatirovsky E, Tuganbaev T, Federici S, Zmora N, Zeevi D, Dori-Bachash M, Pevsner-Fischer M, Kartvelishvily E, Brandis A, Harmelin A, Shibolet O, Halpern Z, Honda K, Amit I, Segal E, Elinav E. 2016. Microbiota diurnal rhythmicity programs host transcriptome oscillations. Cell 167:1495–1510. doi:10.1016/j.cell.2016.11.003 27912059

[11] Zheng D, Ratiner K, Elinav E. 2020. Circadian influences of diet on the microbiome and immunity. Trends Immunol 41:512–530. doi:10.1016/j.it.2020.04.005 32359722

[12] Brooks JF II, Behrendt CL, Ruhn KA, Lee S, Raj P, Takahashi JS, Hooper LV. 2021. The microbiota coordinates diurnal rhythms in innate immunity with the circadian clock. Cell 184:4154–4167. doi:10.1016/j.cell.2021.07.001 34324837 PMC8967342

[13] Graef FA, Celiberto LS, Allaire JM, Kuan MTY, Bosman ES, Crowley SM, Yang H, Chan JH, Stahl M, Yu H, Quin C, Gibson DL, Verdu EF, Jacobson K, Vallance BA. 2021. Fasting increases microbiome-based colonization resistance and reduces host inflammatory responses during an enteric bacterial infection. PLoS Pathog 17:e1009719. doi:10.1371/journal.ppat.1009719 34352037 PMC8341583

[14] Martens EC, Chiang HC, Gordon JI. 2008. Mucosal glycan foraging enhances fitness and transmission of a saccharolytic human gut bacterial symbiont. Cell Host Microbe 4:447–457. doi:10.1016/j.chom.2008.09.007 18996345 PMC2605320

[15] Donaldson GP, Lee SM, Mazmanian SK. 2016. Gut biogeography of the bacterial microbiota. Nat Rev Microbiol 14:20–32. doi:10.1038/nrmicro3552 26499895 PMC4837114

[16] Potrykus K, Cashel M. 2008. (p)ppGpp: still magical? Annu Rev Microbiol 62:35–51. doi:10.1146/annurev.micro.62.081307.162903 18454629

[17] Yi H, Kim HS. 2018. Antibiotic scars left on the gut microbiota from the stringent response. Trends Microbiol 26:735–737. doi:10.1016/j.tim.2018.06.003 30025977

[18] Irving SE, Choudhury NR, Corrigan RM. 2021. The stringent response and physiological roles of (pp)pGpp in bacteria. Nat Rev Microbiol 19:256–271. doi:10.1038/s41579-020-00470-y 33149273

[19] Dalebroux ZD, Svensson SL, Gaynor EC, Swanson MS. 2010. Swanson, ppGpp conjures bacterial virulence. Microbiol Mol Biol Rev 74:171–199. doi:10.1128/MMBR.00046-09 20508246 PMC2884408

[20] Traxler MF, Summers SM, Nguyen H-T, Zacharia VM, Hightower GA, Smith JT, Conway T. 2008. The global, ppGpp-mediated stringent response to amino acid starvation in Escherichia coli. Mol Microbiol 68:1128–1148. doi:10.1111/j.1365-2958.2008.06229.x 18430135 PMC3719176

[21] Sanchez-Vazquez P, Dewey CN, Kitten N, Ross W, Gourse RL. 2019. Genome-wide effects on Escherichia coli transcription from ppGpp binding to its two sites on RNA polymerase. Proc Natl Acad Sci U S A 116:8310–8319. doi:10.1073/pnas.1819682116 30971496 PMC6486775

[22] Kriel A, Brinsmade SR, Tse JL, Tehranchi AK, Bittner AN, Sonenshein AL, Wang JD. 2014. GTP dysregulation in Bacillus subtilis cells lacking (p)ppGpp results in phenotypic amino acid auxotrophy and failure to adapt to nutrient downshift and regulate biosynthesis genes. J Bacteriol 196:189–201. doi:10.1128/JB.00918-13 24163341 PMC3911124

[23] Glass TL, Holmes WM, Hylemon PB, Stellwag EJ. 1979. Synthesis of guanosine tetra- and pentaphosphates by the obligately anaerobic bacterium Bacteroides thetaiotaomicron in response to molecular oxygen. J Bacteriol 137:956–962. doi:10.1128/jb.137.2.956-962.1979 422517 PMC218380

[24] Schofield WB, Zimmermann-Kogadeeva M, Zimmermann M, Barry NA, Goodman AL. 2018. The stringent response determines the ability of a commensal bacterium to survive starvation and to persist in the gut. Cell Host Microbe 24:120–132. doi:10.1016/j.chom.2018.06.002 30008292 PMC6086485

[25] Subramanian A, Tamayo P, Mootha VK, Mukherjee S, Ebert BL, Gillette MA, Paulovich A, Pomeroy SL, Golub TR, Lander ES, Mesirov JP. 2005. Gene set enrichment analysis: a knowledge-based approach for interpreting genome-wide expression profiles. Proc Natl Acad Sci U S A 102:15545–15550. doi:10.1073/pnas.0506580102 16199517 PMC1239896

[26] Kanehisa M, Furumichi M, Sato Y, Kawashima M, Ishiguro-Watanabe M. 2023. KEGG for taxonomy-based analysis of pathways and genomes. Nucleic Acids Res 51:D587–D592. doi:10.1093/nar/gkac963 36300620 PMC9825424

[27] Durfee T, Hansen AM, Zhi H, Blattner FR, Jin DJ. 2008. Transcription profiling of the stringent response in Escherichia coli. J Bacteriol 190:1084–1096. doi:10.1128/JB.01092-07 18039766 PMC2223561

[28] Corrigan RM, Bellows LE, Wood A, Gründling A. 2016. Grundling, ppGpp negatively impacts ribosome assembly affecting growth and antimicrobial tolerance in Gram-positive bacteria. Proc Natl Acad Sci U S A 113:E1710–9. doi:10.1073/pnas.1522179113 26951678 PMC4812758

[29] Kriel A, Bittner AN, Kim SH, Liu K, Tehranchi AK, Zou WY, Rendon S, Chen R, Tu BP, Wang JD. 2012. Direct regulation of GTP homeostasis by (p)ppGpp: a critical component of viability and stress resistance. Mol Cell 48:231–241. doi:10.1016/j.molcel.2012.08.009 22981860 PMC3483369

[30] Ihara Y, Ohta H, Masuda S. 2015. A highly sensitive quantification method for the accumulation of alarmone ppGpp in Arabidopsis thaliana using UPLC-ESI-qMS/MS. J Plant Res 128:511–518. doi:10.1007/s10265-015-0711-1 25752614

[31] Varik V, Oliveira SRA, Hauryliuk V, Tenson T. 2017. HPLC-based quantification of bacterial housekeeping nucleotides and alarmone messengers ppGpp and pppGpp. Sci Rep 7:11022. doi:10.1038/s41598-017-10988-6 28887466 PMC5591245

[32] Fernández-Coll L, Maciag-Dorszynska M, Tailor K, Vadia S, Levin PA, Szalewska-Palasz A, Cashel M. 2020. The absence of (p)ppGpp renders initiation of Escherichia coli chromosomal DNA synthesis independent of growth rates. mBio 11:e03223-19. doi:10.1128/mBio.03223-19 32156825 PMC7064777

[33] Wang JD, Sanders GM, Grossman AD. 2007. Nutritional control of elongation of DNA replication by (p)ppGpp. Cell 128:865–875. doi:10.1016/j.cell.2006.12.043 17350574 PMC1850998

[34] Kraemer JA, Sanderlin AG, Laub MT. 2019. The stringent response inhibits DNA replication initiation in E. coli by modulating supercoiling of oriC. mBio 10:e01330-19. doi:10.1128/mBio.01330-19 31266875 PMC6606810

[35] Korem T, Zeevi D, Suez J, Weinberger A, Avnit-Sagi T, Pompan-Lotan M, Matot E, Jona G, Harmelin A, Cohen N, Sirota-Madi A, Thaiss CA, Pevsner-Fischer M, Sorek R, Xavier R, Elinav E, Segal E. 2015. Growth dynamics of gut microbiota in health and disease inferred from single metagenomic samples. Science 349:1101–1106. doi:10.1126/science.aac4812 26229116 PMC5087275

[36] Haugan MS, Charbon G, Frimodt-Møller N, Løbner-Olesen A. 2018. Chromosome replication as a measure of bacterial growth rate during Escherichia coli infection in the mouse peritonitis model. Sci Rep 8:14961. doi:10.1038/s41598-018-33264-7 30297723 PMC6175860

[37] Glenwright AJ, Pothula KR, Bhamidimarri SP, Chorev DS, Baslé A, Firbank SJ, Zheng H, Robinson CV, Winterhalter M, Kleinekathöfer U, Bolam DN, van den Berg B. 2017. Structural basis for nutrient acquisition by dominant members of the human gut microbiota. Nature 541:407–411. doi:10.1038/nature20828 28077872 PMC5497811

[38] Martens EC, Koropatkin NM, Smith TJ, Gordon JI. 2009. Complex glycan catabolism by the human gut microbiota: the bacteroidetes sus-like paradigm. J Biol Chem 284:24673–24677. doi:10.1074/jbc.R109.022848 19553672 PMC2757170

[39] Pudlo NA, Urs K, Kumar SS, German JB, Mills DA, Martens EC. 2015. Symbiotic human gut bacteria with variable metabolic priorities for host mucosal glycans. mBio 6:e01282–15. doi:10.1128/mBio.01282-15 26556271 PMC4659458

[40] Terrapon N, Lombard V, Drula É, Lapébie P, Al-Masaudi S, Gilbert HJ, Henrissat B. 2018. PULDB: the expanded database of polysaccharide utilization loci. Nucleic Acids Res 46:D677–D683. doi:10.1093/nar/gkx1022 29088389 PMC5753385

[41] Schwalm ND, Townsend GE, Groisman EA. 2017. Prioritization of polysaccharide utilization and control of regulator activation in Bacteroides thetaiotaomicron. Mol Microbiol 104:32–45. doi:10.1111/mmi.13609 28009067

[42] Roghanian M, Semsey S, Løbner-Olesen A, Jalalvand F. 2019. (p)ppGpp-mediated stress response induced by defects in outer membrane biogenesis and ATP production promotes survival in Escherichia coli. Sci Rep 9:2934. doi:10.1038/s41598-019-39371-3 30814571 PMC6393671

[43] Tarusawa T, Ito S, Goto S, Ushida C, Muto A, Himeno H. 2016. (p)ppGpp-dependent and -independent pathways for salt tolerance in Escherichia coli. J Biochem 160:19–26. doi:10.1093/jb/mvw008 26823481

[44] Xu J, Tozawa Y, Lai C, Hayashi H, Ochi K. 2002. A rifampicin resistance mutation in the rpoB gene confers ppGpp-independent antibiotic production in Streptomyces coelicolor A3(2). Mol Genet Genomics 268:179–189. doi:10.1007/s00438-002-0730-1 12395192

[45] Brockmann-Gretza O, Kalinowski J. 2006. Global gene expression during stringent response in Corynebacterium glutamicum in presence and absence of the rel gene encoding (p)ppGpp synthase. BMC Genomics 7:230. doi:10.1186/1471-2164-7-230 16961923 PMC1578569

[46] Potrykus K, Thomas NE, Bruhn-Olszewska B, Sobala M, Dylewski M, James T, Cashel M. 2020. Estimates of Rel(Seq), Mesh1, and SAH(Mex) hydrolysis of (p)ppGpp and (p)ppApp by thin layer chromatography and NADP/NADH coupled assays. Front Microbiol 11:581271. doi:10.3389/fmicb.2020.581271 33193211 PMC7644958

[47] Sun Z, Wu R, Zhao B, Zeinert R, Chien P, You M. 2021. Live-cell imaging of guanosine tetra- and pentaphosphate (p)ppGpp with RNA-based fluorescent sensors*. Angew Chem Int Ed Engl 60:24070–24074. doi:10.1002/anie.202111170 34487413 PMC8545912

[48] Meyer L, Germain E, Maisonneuve E. 2021. Regulation of ytfK by cAMP-CRP contributes to SpoT-dependent accumulation of (p)ppGpp in response to carbon starvation YtfK responds to glucose exhaustion. Front Microbiol 12:775164. doi:10.3389/fmicb.2021.775164 34803996 PMC8600398

[49] Kitazawa T, Kaiya H. 2019. Regulation of gastrointestinal motility by Motilin and Ghrelin in vertebrates. Front Endocrinol (Lausanne) 10:278. doi:10.3389/fendo.2019.00278 31156548 PMC6533539

[50] Ratiner K, Fachler-Sharp T, Elinav E. 2023. Small intestinal microbiota oscillations, host effects and regulation-A zoom into three key effector molecules. Biology (Basel) 12:142. doi:10.3390/biology12010142 36671834 PMC9855434

[51] Penny HA, Domingues RG, Krauss MZ, Melo-Gonzalez F, Lawson MAE, Dickson S, Parkinson J, Hurry M, Purse C, Jegham E, Godinho-Silva C, Rendas M, Veiga-Fernandes H, Bechtold DA, Grencis RK, Toellner K-M, Waisman A, Swann JR, Gibbs JE, Hepworth MR. 2022. Rhythmicity of intestinal IgA responses confers oscillatory commensal microbiota mutualism. Sci Immunol 7:eabk2541. doi:10.1126/sciimmunol.abk2541 36054336 PMC7613662

[52] Tuganbaev T, Mor U, Bashiardes S, Liwinski T, Nobs SP, Leshem A, Dori-Bachash M, Thaiss CA, Pinker EY, Ratiner K, et al.. 2020. Diet diurnally regulates small intestinal microbiome-epithelial-immune homeostasis and enteritis. Cell 182:1441–1459. doi:10.1016/j.cell.2020.08.027 32888430

[53] Talbot J, Hahn P, Kroehling L, Nguyen H, Li D, Littman DR. 2020. Feeding-dependent VIP neuron-ILC3 circuit regulates the intestinal barrier. Nature 579:575–580. doi:10.1038/s41586-020-2039-9 32050257 PMC7135938

[54] Cui Y, Li S, Yin Y, Li X, Li X. 2022. Daytime restricted feeding promotes circadian desynchrony and metabolic disruption with changes in bile acids profiles and gut microbiota in C57BL/6 male mice. J Nutr Biochem 109:109121. doi:10.1016/j.jnutbio.2022.109121 35940511

[55] Opperhuizen A-L, Wang D, Foppen E, Jansen R, Boudzovitch-Surovtseva O, de Vries J, Fliers E, Kalsbeek A. 2016. Feeding during the resting phase causes profound changes in physiology and desynchronization between liver and muscle rhythms of rats. Eur J Neurosci 44:2795–2806. doi:10.1111/ejn.13377 27562056

[56] Froy O, Chapnik N, Miskin R. 2009. Effect of intermittent fasting on circadian rhythms in mice depends on feeding time. Mech Ageing Dev 130:154–160. doi:10.1016/j.mad.2008.10.006 19041664

[57] Mukherji Atish, Kobiita A, Damara M, Misra N, Meziane H, Champy M-F, Chambon P. 2015. Shifting eating to the circadian rest phase misaligns the peripheral clocks with the master SCN clock and leads to a metabolic syndrome. Proc Natl Acad Sci U S A 112:E6691–8. doi:10.1073/pnas.1519807112 26627260 PMC4672793

[58] Mukherji A., Kobiita A, Chambon P. 2015. Shifting the feeding of mice to the rest phase creates metabolic alterations, which, on their own, shift the peripheral circadian clocks by 12 hours. Proc Natl Acad Sci U S A 112:E6683–90. doi:10.1073/pnas.1519735112 26627259 PMC4672831

[59] Wang B, Dai P, Ding D, Del Rosario A, Grant RA, Pentelute BL, Laub MT. 2019. Affinity-based capture and identification of protein effectors of the growth regulator ppGpp. Nat Chem Biol 15:141–150. doi:10.1038/s41589-019-0296-4 30559427 PMC6366861

[60] Zhang Y, Zborníková E, Rejman D, Gerdes K, Swanson MS. 2018. Novel (p)ppGpp binding and metabolizing proteins of Escherichia coli. mBio 9:e02188-17. doi:10.1128/mBio.02188-17 29511080 PMC5845004

[61] Liu K, Myers AR, Pisithkul T, Claas KR, Satyshur KA, Amador-Noguez D, Keck JL, Wang JD. 2015. Molecular mechanism and evolution of guanylate kinase regulation by (p)ppGpp. Mol Cell 57:735–749. doi:10.1016/j.molcel.2014.12.037 25661490 PMC4336630

[62] Anderson BW, Liu K, Wolak C, Dubiel K, She F, Satyshur KA, Keck JL, Wang JD. 2019. Evolution of (p)ppGpp-HPRT regulation through diversification of an allosteric oligomeric interaction. Elife 8:e47534. doi:10.7554/eLife.47534 31552824 PMC6783271

[63] Haseltine WA, Block R. 1973. Synthesis of guanosine tetra- and pentaphosphate requires the presence of a codon-specific, uncharged transfer ribonucleic acid in the acceptor site of ribosomes. Proc Natl Acad Sci U S A 70:1564–1568. doi:10.1073/pnas.70.5.1564 4576025 PMC433543

[64] Loveland AB, Bah E, Madireddy R, Zhang Y, Brilot AF, Grigorieff N, Korostelev AA. 2016. Ribosome center DOT rela structures reveal the mechanism of stringent response activation. Elife 5:e17029. doi:10.7554/eLife.17029 27434674 PMC4974054

[65] Battesti A, Bouveret E. 2006. Acyl carrier protein/SpoT interaction, the switch linking SpoT-dependent stress response to fatty acid metabolism. Mol Microbiol 62:1048–1063. doi:10.1111/j.1365-2958.2006.05442.x 17078815

[66] Seyfzadeh M, Keener J, Nomura M. 1993. spoT-dependent accumulation of guanosine tetraphosphate in response to fatty acid starvation in Escherichia coli. Proc Natl Acad Sci U S A 90:11004–11008. doi:10.1073/pnas.90.23.11004 7504290 PMC47910

[67] Germain E, Guiraud P, Byrne D, Douzi B, Djendli M, Maisonneuve E. 2019. YtfK activates the stringent response by triggering the alarmone synthetase SpoT in Escherichia coli. Nat Commun 10:5763. doi:10.1038/s41467-019-13764-4 31848343 PMC6917717

[68] Pizarro-Cerdá J, Tedin K. 2004. The bacterial signal molecule, ppGpp, regulates Salmonella virulence gene expression. Mol Microbiol 52:1827–1844. doi:10.1111/j.1365-2958.2004.04122.x 15186428

[69] Abranches J, Martinez AR, Kajfasz JK, Chávez V, Garsin DA, Lemos JA. 2009. The molecular alarmone (p)ppGpp mediates stress responses, vancomycin tolerance, and virulence in Enterococcus faecalis. J Bacteriol 191:2248–2256. doi:10.1128/JB.01726-08 19168608 PMC2655485

[70] Das B, Bhadra RK. 2020. (p)ppGpp metabolism and antimicrobial resistance in bacterial pathogens. Front Microbiol 11:563944. doi:10.3389/fmicb.2020.563944 33162948 PMC7581866

[71] Hutchison AT, Regmi P, Manoogian ENC, Fleischer JG, Wittert GA, Panda S, Heilbronn LK. 2019. Time-restricted feeding improves glucose tolerance in men at risk for type 2 diabetes: a randomized crossover trial. Obesity (Silver Spring) 27:724–732. doi:10.1002/oby.22449 31002478

[72] Sutton EF, Beyl R, Early KS, Cefalu WT, Ravussin E, Peterson CM. 2018. Early time-restricted feeding improves insulin sensitivity, blood pressure, and oxidative stress even without weight loss in men with prediabetes. Cell Metab 27:1212–1221. doi:10.1016/j.cmet.2018.04.010 29754952 PMC5990470

[73] Shirasu-Hiza MM, Dionne MS, Pham LN, Ayres JS, Schneider DS. 2007. Interactions between circadian rhythm and immunity in Drosophila melanlogaster. Curr Biol 17:R353–5. doi:10.1016/j.cub.2007.03.049 17502084

[74] Pollmächer T, Mullington J, Korth C, Schreiber W, Hermann D, Orth A, Galanos C, Holsboer F. 1996. Diurnal variations in the human host response to endotoxin. J Infect Dis 174:1040–1045. doi:10.1093/infdis/174.5.1040 8896506

[75] Cullen TW, Schofield WB, Barry NA, Putnam EE, Rundell EA, Trent MS, Degnan PH, Booth CJ, Yu H, Goodman AL. 2015. Antimicrobial peptide resistance mediates resilience of prominent gut commensals during inflammation. Science 347:170–175. doi:10.1126/science.1260580 25574022 PMC4388331

[76] Koropatkin NM, Martens EC, Gordon JI, Smith TJ. 2008. Starch catabolism by a prominent human gut symbiont is directed by the recognition of amylose helices. Structure 16:1105–1115. doi:10.1016/j.str.2008.03.017 18611383 PMC2563962

[77] Langmead B, Salzberg SL. 2012. Fast gapped-read alignment with Bowtie 2. Nat Methods 9:357–359. doi:10.1038/nmeth.1923 22388286 PMC3322381

[78] Liao Y, Smyth GK, Shi W. 2014. Shi, featureCounts: an efficient general purpose program for assigning sequence reads to genomic features. Bioinformatics 30:923–930. doi:10.1093/bioinformatics/btt656 24227677

[79] Love MI, Huber W, Anders S. 2014. Moderated estimation of fold change and dispersion for RNA-seq data with DESeq2. Genome Biol 15:550. doi:10.1186/s13059-014-0550-8 25516281 PMC4302049

[80] Wickham H. 2016. ggplot2: elegant graphics for data analysis. Springer, Cham.

[81] Rana S, Blighe K, Lewis M. 2023. EnhancedVolcano: publication-ready volcano plots with enhanced colouring and labeling

[82] Emiola A, Oh J. 2018. High throughput in situ metagenomic measurement of bacterial replication at ultra-low sequencing coverage. Nat Commun 9:4956. doi:10.1038/s41467-018-07240-8 30470746 PMC6251912

