# Supplementary material for: Gut microbes modulate (p)ppGpp during a time-restricted feeding regimen
Source: mBio. 2023 Nov 16;14(6):e01907-23. doi: 10.1128/mbio.01907-23 (PMC10746209; doi:10.1128/mbio.01907-23)
Supplement: Supplemental Legends — Legends for supplemental figures and tables. [file mbio.01907-23-s0006.docx]

**Supplementary Tables and Figures**

**Fig. S1. Limited overlap between gene expression changes in *B. thetaiotaomicron* WT and (p)ppGpp^0^ strains during fed and fasted phases in mice.** A) Venn diagram of significantly differentially regulated genes between fed and fasted states in the WT and (p)ppGpp^0^ strains. Significance was defined using a false discovery rate (FDR) of < 0.05 and a log_2_(FoldChange) > 1 or < −1. B) The majority of gene expression changes shared between WT and (p)ppGpp^0^ strains reflect genes upregulated in the fed state.

**Fig. S2. Representative chromatograms used in ppGpp quantification.** A) Overview of the ppGpp isolation workflow. B) Chromatogram traces (left column) and UV-visible spectra (right column) of extracted cecal contents from gnotobiotic mice colonized with *B. thetaiotaomicron* WT (top panel) or (p)ppGpp^0^ strains (middle and bottom panels). In the bottom panel, 400 pmol ppGpp standard was spiked into cecal contents from *B. thetaiotaomicron* (p)ppGpp^0^-monoassociated mice before extraction.

**Fig. S3. *B. thetaiotaomicron* GRiD values in varying growth conditions *in vitro*.** While both WT and (p)ppGpp^0^ strains reduce GRiD values in response to nutrient limitation (stationary phase or 1-hour carbon starvation), the magnitude of the response is greater in the WT strain. The (p)ppGpp^0^ strain exhibits a lower GRiD value compared to the WT strain in the mid-log TYG condition, potentially due to undefined components of this medium that are poorly utilized by the (p)ppGpp^0^ strain. The GRiD values of the WT strain in this condition are the highest observed across the tested conditions (and significantly higher than the values from *in vivo* conditions); the (p)ppGpp^0^ strain may be unable to achieve this maximal replication rate that can be produced under these *in vitro* conditions. Consistent with both of these possibilities, both strains have similar GRiD values in glucose minimal medium, which constrains the WT strain to replication rates that are more similar to *in vivo* conditions. Notably, the WT and ppGpp^0^ strains have similar growth rates in the mid-log TYG condition, suggesting that the replication rate at these very high GRiD values is not the limiting factor determining growth rate. “Glucose” is abbreviated as “Glu”. Significance is indicated with asterisks (*p < 0.05; **p < 0.01; ***p < 0.001, ****p<0.0001 ANOVA followed by Tukey HSD post-hoc test).

**Figure S4. Transcriptional responses of *B. thetaiotaomicron* WT and (p)ppGpp^0^ strains during diauxic growth in PGA and PMOG.** A) Growth of WT and (p)ppGpp^0^ strains in 0.2% PGA and 0.6% PMOG. Arrows indicate timepoints selected for analysis of gene expression of PGA- and PMOG-responsive PULs (Pudlo et al. 2015). B) Differential expression (early timepoint vs. late timepoint) of genes encoded in PGA- and PMOG-responsive PULs in B. thetaiotaomicron WT and (p)ppGpp^0^ strains. PUL number and predicted substrates are from PULDB (Terrapon et al., 2018). Significance is indicated with asterisks (*p < 0.05; **p < 0.01; unpaired t-test).

**Figure S5. Doubling time of independently constructed *B. thetaiotaomicron* (p)ppGpp^0^ strains in minimal medium containing PGA and PMOG (0.2%:0.6%).** Doubling times were calculated between OD_600_ ~0.2-0.4 (before diauxic shift) and during the two hour period corresponding to the second exponential phase in the WT strain (after diauxic shift).

**Table S1.** RPKM values of (p)ppGpp^0^ and WT strains isolated from cecal contents of fed and fasted mice, related to Figure 1 and 2.

**Table S2.** Primers used for qRT-PCR, related to figure S4.
